# ERCC6L in human cancers: oncogenic functions, molecular mechanisms, and clinical implications as a prognostic biomarker and therapeutic target

**DOI:** 10.3389/fonc.2026.1812829

**Published:** 2026-07-01

**Authors:** Lingyu Jiang, Huaihai Zhou, Jing He, Junqi Qin, Jianwei Huang, Jiaping Wei, Yifan Zhou, Yonglong Zhong

**Affiliations:** 1 Intensive Care Unit, The People’s Hospital of Guangxi Zhuang Autonomous Region, Guangxi Academy of Medical Sciences, Nanning, China; 2 Department of Respiratory and Critical Care, The People’s Hospital of Guangxi Zhuang Autonomous Region, Guangxi Academy of Medical Sciences, Nanning, China; 3 Department of Thoracic Surgery, The People’s Hospital of Guangxi Zhuang Autonomous Region, Guangxi Academy of Medical Sciences, Nanning, China

**Keywords:** biomarker, carcinogenesis, ERCC6L, oncogene, signal transduction

## Abstract

Excision repair cross-complementation group 6-like (ERCC6L), also known as PICH, is a centromere-associated SNF2 family ATPase that functions as a DNA helicase essential for mitotic chromosome segregation. Emerging evidence has established its significant oncogenic role across a broad spectrum of human malignancies. This review synthesizes current knowledge on the expression landscape, oncogenic functions, molecular mechanisms, and clinical significance of ERCC6L in cancer. Pan-cancer analyses consistently demonstrate that ERCC6L is frequently overexpressed in most tumor types compared to normal tissues, driven by mechanisms such as DNA amplification and promoter hypomethylation. This upregulation strongly correlates with aggressive clinicopathological features, including advanced tumor stage, metastasis, and poor patient prognosis across cancers like breast cancer, hepatocellular carcinoma, lung adenocarcinoma, and gastric cancer. Functionally, ERCC6L acts as a potent driver of malignant phenotypes by promoting uncontrolled proliferation through cell cycle acceleration, exerting anti-apoptotic effects, and enhancing invasion and metastasis via epithelial-mesenchymal transition. Mechanistically, ERCC6L operates at the nexus of multiple cancer pathways. It interacts with key mitotic regulators including PLK1, FOXM1, and KIF4A to govern cell cycle progression, activates pro-survival signaling cascades such as PI3K/AKT and NF-κB, and drives metastasis via PJA2-mediated p53 ubiquitination in lung adenocarcinoma. Furthermore, ERCC6L promotes metabolic reprogramming by stabilizing HIF-1α or transactivating PLK1 to drive aerobic glycolysis, and modulates DNA damage response, linking it to radio- and chemoresistance. Emerging evidence also connects ERCC6L to an immunosuppressive tumor microenvironment, with associations including Th2 cell infiltration, macrophage polarization, and reduced immune infiltration in HRD-high tumors, suggesting potential as a predictive biomarker for immunotherapy. The consistent association with aggressive tumor behavior positions ERCC6L as a valuable prognostic biomarker and a promising therapeutic target. Preclinical studies demonstrate that ERCC6L inhibition suppresses tumor growth and enhances treatment efficacy; however, current evidence is limited to *in vitro* and xenograft models, and substantial further validation is required before clinical translation.

## Introduction

Excision repair cross-complementation group 6-like (ERCC6L), also known as PICH, is a centromere-associated SNF2 family ATPase that functions as a DNA helicase and translocase (1). The dual nomenclature reflects its genetic origin (ERCC6L, Excision Repair Cross-Complementing Group 6 Like) and, more functionally, its identity as a Polo-like kinase 1−interacting checkpoint helicase (PICH) that associates with kinetochores and resolves ultrafine anaphase DNA bridges to ensure genomic fidelity (1, 2) [1]. Beyond its fundamental cellular function in chromosome segregation, ERCC6L was initially identified as a crucial factor in embryonic development and teratogenic responses. Early studies using *in situ* hybridization and Northern blotting demonstrated that Ercc6l is expressed in the embryonic neural tube and heart, and that alcohol exposure significantly downregulates its expression in these organs, suggesting a role in the teratogenic action of alcohol (3). Subsequently, Yin et al. successfully cloned the full-length Ercc6l cDNA from Sika deer and confirmed by qRT-PCR that Ercc6l transcripts are present in virtually all examined organs across multiple developmental stages, further supporting a conserved function of ERCC6L in mammalian development (4). More recently, PICH has been implicated in the reprogramming of induced pluripotent stem cells (iPSCs), where it alleviates genomic instability caused by DNA replication stress, further expanding the functional repertoire of this helicase beyond mitosis (5). Emerging evidence has consistently implicated ERCC6L in the pathogenesis of human malignancies.

Pu et al. (6) first systematically investigated the transition from an embryonic regulator to an oncogenic driver, demonstrating that ERCC6L is overexpressed across 12 solid tumor types, its knockdown inhibits cell proliferation by reducing MAPK and CDK2 expression through the downregulation of RAB31, its deficiency triggers apoptosis, and its high expression is associated with worse clinical survival in breast and kidney cancers. Initially identified through transcriptomic analyses as a gene frequently overexpressed in solid tumors (6), subsequent research has progressively unveiled its significant oncogenic potential across a wide spectrum of cancers.

The deregulation of DNA repair and cell cycle control mechanisms represents a hallmark of cancer. Given its intrinsic involvement in these critical processes, ERCC6L has naturally become a focus of cancer research. Early studies established a correlation between high ERCC6L expression and poor patient outcomes in specific cancers such as breast and kidney tumors (6). This initial observation paved the way for more extensive investigations that now span multiple cancer types. Recent pan-cancer analyses corroborate that ERCC6L mRNA levels are elevated in the majority of cancer types compared to their corresponding normal tissues, and this upregulation is frequently associated with advanced disease stages and unfavorable prognosis (7). The consistent pattern of dysregulation suggests that ERCC6L is not merely a passive bystander but an active contributor to the malignant phenotype.

A growing body of functional studies supports an oncogenic role for ERCC6L at multiple stages of tumor development. Silencing ERCC6L consistently inhibits cancer cell proliferation, colony formation, migration, and invasion (6, 8–11), while its overexpression exerts the opposite effects, accelerating tumor growth *in vitro* and *in vivo* (8, 10). Mechanistically, ERCC6L intersects with several core cancer-related signaling pathways, influencing cell cycle transitions (8), DNA damage response (12), apoptosis (6, 11, 13), and metabolic reprogramming (14, 15). Its interaction with key mitotic regulators like PLK1 (8, 15) and KIF4A (8) further underscores its importance in maintaining the proliferative drive of cancer cells. This review aims to synthesize the current knowledge on ERCC6L, elucidating its expression landscape, multifaceted oncogenic functions, underlying molecular mechanisms, and its promising potential as a prognostic biomarker and therapeutic target in human cancers.

## Literature search strategy

This narrative review was conducted following a structured literature search to ensure a comprehensive and transparent synthesis of the available evidence. We systematically searched the PubMed and Web of Science databases for articles published up to May 2026. The search strategy combined keywords related to the target gene (ERCC6L, PICH, excision repair cross-complementation group 6-like, PLK1-interacting checkpoint helicase) and the condition (cancer, tumor, carcinoma, neoplasm, malignancy, oncology). The search was limited to English-language publications and human studies. We included original articles, systematic reviews, meta-analyses, and major case series reporting on ERCC6L expression, functional characterization, or clinical associations in human cancers. Reference lists of included articles were manually screened to identify additional relevant studies. The literature identification, screening, eligibility assessment, and inclusion process is presented in **Supplementary Figure 1**. Given the narrative nature of this review, we did not perform a formal quality assessment or meta-analysis. The selection of studies was based on their relevance to the key themes of the review: expression landscape, oncogenic functions, molecular mechanisms, and clinical implications as a prognostic biomarker and therapeutic target.

## Expression landscape and clinical significance of ERCC6L across human cancers

A consistent and prominent finding across numerous studies is the widespread upregulation of ERCC6L in human malignancies. Pan-cancer bioinformatics analyses utilizing large-scale datasets like The Cancer Genome Atlas (TCGA) have provided a comprehensive overview. One such analysis by Lu et al., which examined 33 tumor types, revealed that ERCC6L mRNA expression is significantly higher in most cancer types compared to adjacent normal tissues and that this overexpression is associated with promoter hypomethylation and a positive correlation with Th2 immune cell infiltration, establishing it as a commonly dysregulated gene in oncogenesis (7). To facilitate cross-cancer comparison, we have summarized the expression status, clinicopathological associations, and prognostic value of ERCC6L in **Table 1**.

**Table 1:** Summary of ERCC6L dysregulation and clinical significance across human cancers.

Cancer type	Expression status	Associated clinicopathological features	Prognostic value	Key molecular mechanisms	References
Breast cancer (especially TNBC)	Upregulated	High grade, HRD status	Poor OS	Cell cycle acceleration (G2/M); KIF4A interaction; MAPK signaling	(6, 8, 12, 13, 16)
Hepatocellular carcinoma (HCC)	Upregulated	High AFP, vascular invasion, advanced stage	Independent poor OS, RFS	PI3K/AKT, NF-κB activation; DNA repair pathway enrichment	(17–19)
Lung adenocarcinoma (LUAD)	Upregulated	Nodal invasion, advanced stage	Independent poor OS, PFI, DSS	Wnt/EMT; HIF-1α-driven glycolysis; PJA2/p53-mediated metastasis	(9, 14, 20, 21)
Gastric cancer (GC)	Upregulated	Larger tumor, advanced stage, macrophage polarization	Poor prognosis	NF-κB signaling; EMT; macrophage polarization/lactylation	(10, 22, 23)
Colorectal cancer (CRC)	Upregulated	Larger tumor	–	G0/G1 cell cycle arrest	(24)
Renal cell carcinoma (RCC)	Upregulated	Higher Fuhrman grade	Poor OS	MAPK signaling; TSC/mTOR pathway	(7, 11)
Laryngeal squamous cell carcinoma (LSCC)	Upregulated	Advanced TNM stage	–	FOXM1-KIF4A feed-forward loop; ROS elevation	(25)
Cutaneous melanoma	Upregulated	Independent poor prognostic factor	Poor survival	PLK1-driven glycolysis; ChIP-confirmed PLK1 activation	(15)
Neuroblastoma	Part of prognostic signatures	4-gene signature; independent OS/EFS predictor	Adverse prognosis	Stemness regulation	(26, 27)

AFP, alpha-fetoprotein; HRD, homologous recombination deficiency; OS, overall survival; RFS, recurrence-free survival; PFI, progression-free interval; DSS, disease-specific survival; ROS, reactive oxygen species.

This overexpression is not uniform but shows particular prominence in certain aggressive subtypes, such as triple-negative breast cancer (TNBC) (8). In breast cancer, multiple studies confirm that high ERCC6L expression correlates with worse overall survival and has been integrated into multi-gene prognostic signatures (6, 16).

The clinical evidence for each cancer type, as summarized in **Table 1**, is discussed in detail below. In hepatocellular carcinoma (HCC), high ERCC6L levels are associated with elevated alpha-fetoprotein (AFP), vascular invasion, advanced histologic grade and TNM stage, and serve as an independent prognostic factor for poorer overall and recurrence-free survival (17). Research also confirms ERCC6L upregulation in HCC and its independent prognostic value (18). Notably, a nomogram combining ERCC6L and TNM stage outperformed TNM stage alone in predicting prognosis (17).

Similarly, in lung adenocarcinoma (LUAD), ERCC6L overexpression is closely linked to nodal invasion, advanced clinical stage, and is an independent biomarker for worse overall survival, progression-free interval, and disease-specific survival (9, 20). This prognostic significance appears subtype-specific within lung cancer, being strongly associated with poor outcomes in LUAD but not necessarily in lung squamous cell carcinoma (LUSC) (20).

The pattern extends to gastrointestinal cancers. In gastric cancer (GC), upregulated ERCC6L correlates with larger tumor size, advanced clinical stage, and poor prognosis (10, 22). ERCC6L has also been identified within gene signatures related to macrophage polarization, further linking it to the tumor microenvironment (22). Colorectal cancer (CRC) tissues and cell lines also exhibit higher ERCC6L levels compared to normal controls, with its expression significantly associated with tumor size (24).

In renal cell carcinoma (RCC), particularly clear cell and chromophobe subtypes, ERCC6L expression increases with higher Fuhrman grade and predicts poor overall survival (11, 28).

Beyond carcinomas, ERCC6L is implicated in other malignancies. Its expression is elevated in laryngeal squamous cell carcinoma (LSCC) and correlates with TNM stage (25). In cutaneous melanoma, ERCC6L is upregulated and drives progression via metabolic reprogramming. Even in neuroblastoma, a pediatric solid tumor, ERCC6L is part of gene signatures predictive of adverse prognosis (26, 27).

The deregulation of ERCC6L expression is governed by multiple genetic and epigenetic mechanisms. DNA amplification and promoter hypomethylation are frequently observed, suggesting regulation at both genetic and epigenetic levels (9, 17). For instance, hypomethylation of the ERCC6L promoter contributes to its overexpression in HCC (17). Furthermore, downregulation of specific miRNAs, such as miR-5589 in HCC, may relieve post-transcriptional repression, leading to ERCC6L accumulation (17). These layered mechanisms ensure sustained high expression of ERCC6L in the tumor microenvironment, facilitating its oncogenic functions. Beyond these intrinsic genetic and epigenetic alterations, the tumor microenvironment itself can modulate ERCC6L expression. For instance, extracellular acidosis—a hallmark of the tumor milieu—has been shown to alter miRNA expression profiles in tumor cells (29) and directly downregulate Ercc6l mRNA and protein levels (30). These findings suggest that ERCC6L expression is dynamically responsive to microenvironmental cues, adding another layer of complexity to its regulation in cancer.

It is important to note that a substantial proportion of the expression and prognostic data summarized above derives from computational analyses of public datasets such as TCGA, which provide correlative rather than causal evidence. In the following sections, we distinguish, where possible, between computationally predicted associations and experimentally confirmed oncogenic mechanisms.

## Oncogenic functions of ERCC6L in tumorigenesis and progression

Having established the widespread overexpression of ERCC6L, we next discuss how this upregulation translates into oncogenic functions. Functional investigations using loss-of-function and gain-of-function approaches have unequivocally established ERCC6L as a potent driver of malignant phenotypes. Its oncogenic activities span the core hallmarks of cancer, primarily fueling cell proliferation, averting cell death, and enabling invasion and metastasis.

The most consistently reported function of ERCC6L is the promotion of uncontrolled cell proliferation. Silencing ERCC6L using siRNA or shRNA significantly inhibits the growth of cancer cells from various origins, including breast (6, 8, 13), liver (18, 19), lung (9, 20), gastric (10), colorectal (24), renal (11), and laryngeal (25) carcinomas. Additionally, in cutaneous melanoma, genetic ablation of ERCC6L significantly inhibits proliferation, migration, and invasion, while promoting apoptosis (15). This anti-proliferative effect is corroborated *in vivo*, where ERCC6L knockdown markedly impedes xenograft tumor growth in immunodeficient mice (6, 11, 19). Conversely, overexpression of ERCC6L accelerates cell proliferation (8, 10). The pro-proliferative effect is largely achieved through the dysregulation of the cell cycle. ERCC6L facilitates cell cycle progression by accelerating the G1/S transition or regulating the G2/M checkpoint, thereby increasing the population of cells in S-phase and reducing those in G0/G1 or G2/M phases (6, 8, 18, 24). This cell cycle acceleration is a fundamental mechanism through which ERCC6L fuels tumor expansion.

Concurrently, ERCC6L exerts anti-apoptotic effects to enhance cell survival. Knockdown of ERCC6L increases the apoptosis rate in various cancer cell lines, such as breast cancer MDA-MB-231 cells and renal cancer 786-O and Caki-1 cells (11, 13). In lung adenocarcinoma cells, ERCC6L silencing promotes apoptosis and leads to cell cycle arrest (9). The perturbation of normal cell cycle checkpoints and the suppression of apoptotic pathways collectively create a permissive environment for the survival and accumulation of genetically unstable cells.

Beyond growth and survival, ERCC6L is a critical facilitator of tumor invasion and metastasis, key drivers of cancer mortality. *In vitro* functional assays demonstrate that reducing ERCC6L expression markedly decreases the migratory and invasive capacities of cancer cells from breast (8), lung (9), gastric (10), colorectal (24), and laryngeal (25) origins. This effect is mediated, in part, through the promotion of epithelial-mesenchymal transition (EMT), a process essential for metastasis. In breast cancer, ERCC6L knockdown inhibits EMT and metastatic behaviors (8). In lung adenocarcinoma, ERCC6L is positively correlated with EMT and may regulate this process through Wnt/β-catenin and Wnt/Notch3 signaling pathways (9). In gastric cancer, ERCC6L promotes metastasis both *in vitro* and *in vivo*, with its knockdown inhibiting these malignant behaviors (10). In laryngeal squamous cell carcinoma, ERCC6L knockdown decreases migration and invasion (25). The ability of ERCC6L to enhance motility and invasion underscores its role in the advanced, lethal stages of cancer progression.

## Molecular mechanisms underlying ERCC6L-driven malignancy

**Figure 1:**
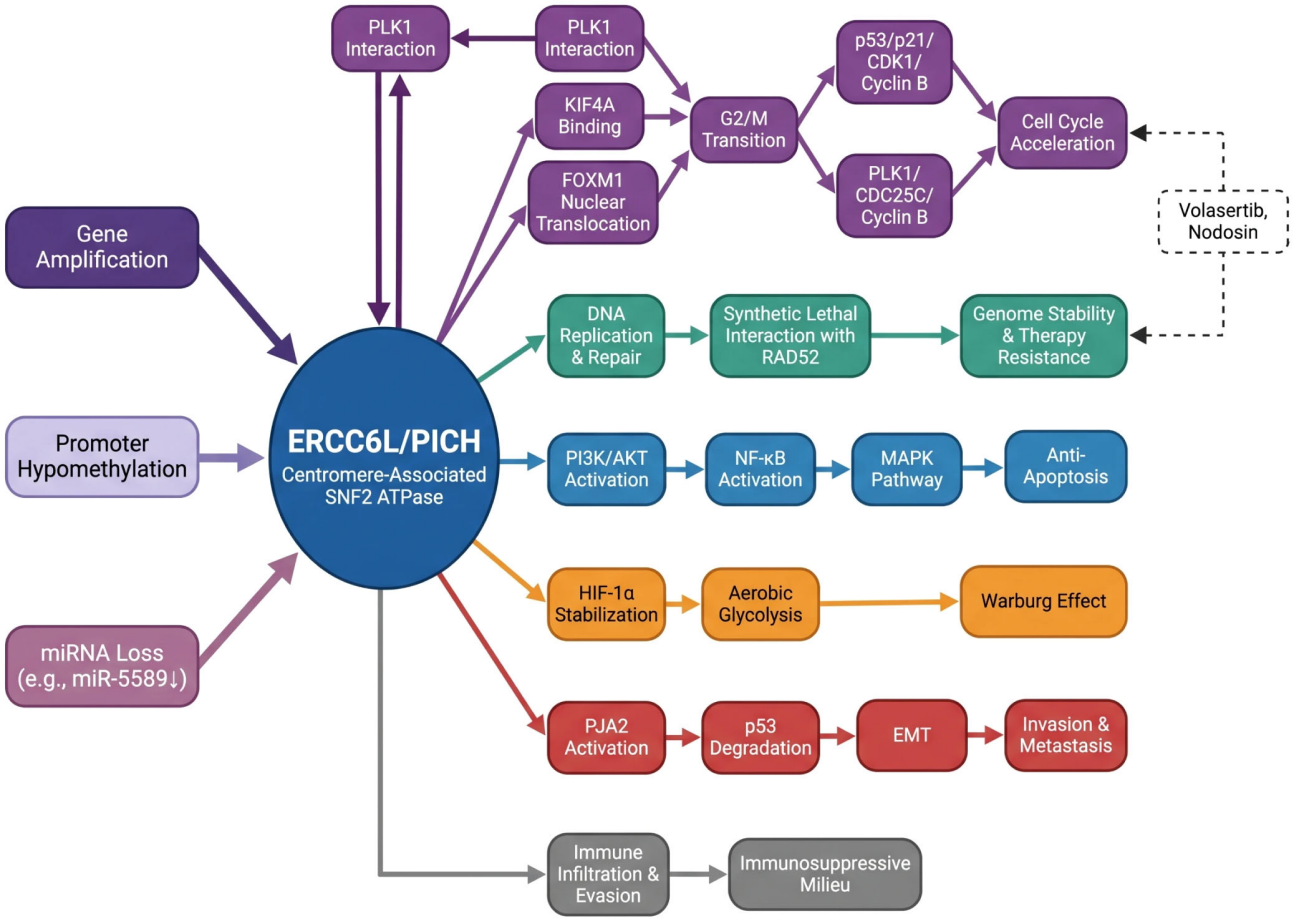
Schematic overview of the ERCC6L-driven oncogenic signaling network. Following upstream activation by gene amplification, promoter hypomethylation, or miRNA loss, ERCC6L/PICH engages multiple downstream pathways: cell cycle acceleration via PLK1/KIF4A/FOXM1 (purple), DNA repair and replication with a synthetic lethal relationship with RAD52 (teal), anti-apoptosis signaling through PI3K/AKT, NF-κB, and MAPK (blue), metabolic reprogramming (Warburg effect) via HIF-1α and PLK1-driven glycolysis (orange), and metastasis through PJA2-mediated p53 degradation and EMT (red). In the tumor microenvironment, ERCC6L correlates with Th2 infiltration, macrophage polarization, and cGAS-STING-mediated immune evasion (gray). Solid Arrow: Activation/Flow; Dashed Arrow: Therapeutic Target/Hypothetical; Double Arrow: Mutual Interaction; Flat - Head Arrow: Inhibition.

The pleiotropic oncogenic effects of ERCC6L are mediated through its interactions with and modulation of several pivotal cellular signaling pathways and biological processes. Mechanistic studies have begun to unravel the complex network through which ERCC6L exerts its tumor-promoting functions. A schematic overview of these pathways is presented in **Figure 1**. In the following, we present the mechanisms organized by the tumor types in which they were primarily elucidated.

### Breast cancer

A central node in ERCC6L’s mechanism of action is its interaction with key regulators of mitosis and the cell cycle. In breast cancer, ERCC6L accelerates the cell cycle by regulating the G2/M transition through modulating of the p53/p21/CDK1/Cyclin B and PLK1/CDC25C/CDK1/Cyclin B axes (8). Furthermore, a direct protein-protein interaction between ERCC6L and kinesin family member 4A (KIF4A) has been identified; both proteins are closely associated with mitosis and collaboratively contribute to breast tumor growth and metastasis (8). Early work also suggested that ERCC6L stimulates proliferation via a RAB31-MAPK-CDK2 pathway (6).

### Hepatocellular carcinoma

In hepatocellular carcinoma, ERCC6L promotes tumor progression by activating both the PI3K/AKT and NF-κB signaling pathways (19). The anti-proliferative effect of the natural compound nodosin on HCC cells is mediated through suppression of the ERCC6L/PI3K/AKT axis (31). Beyond activating PI3K/AKT and NF-κB cascades, ERCC6L has been implicated in sustaining the DNA metabolic demands of highly proliferative cancer cells. In HCC, GSEA revealed that ERCC6L upregulation is significantly associated with the enrichment of DNA replication, homologous recombination, mismatch repair, and excision repair pathways (17). In LUAD, co-expression analyses similarly identified strong functional correlations with cell cycle, DNA damage, and DNA repair pathways (9). These convergent findings from independent studies indicate that ERCC6L plays a conserved role in coordinating DNA repair and replication signaling to support tumor progression.

### Lung adenocarcinoma

In LUAD, PICH deficiency was shown to cause excessive DNA damage and chromosomal instability *in vivo*, directly linking ERCC6L loss to genomic instability and tumor suppression (32). In lung adenocarcinoma, ERCC6L’s functions are positively correlated with DNA damage and repair pathways, and it may regulate EMT through Wnt signaling (9). Emerging evidence shows that ERCC6L enhances glycolysis and stemness by stabilizing hypoxia-inducible factor-1α (HIF-1α) through inhibiting its hydroxylation and ubiquitin-mediated degradation, thereby upregulating key glycolytic enzymes (14). In addition to metabolic reprogramming, a recent study from our group has uncovered a novel mechanism by which ERCC6L drives LUAD metastasis independently of its canonical mitotic roles. ERCC6L was found to activate the E3 ubiquitin ligase PJA2, which catalyzes K48-linked polyubiquitination and subsequent proteasomal degradation of p53, thereby attenuating its tumor-suppressive functions. *In vivo* experiments confirmed that ERCC6L knockout suppressed tumor growth, metastasis, and EMT progression through regulation of the PJA2/p53 signaling axis, identifying this pathway as a potential therapeutic target for inhibiting LUAD metastasis (21).

### Gastric cancer

In gastric cancer, ERCC6L drives malignant phenotypes through NF-κB activation. These effects are accompanied by EMT marker alterations, including E-cadherin downregulation and N-cadherin upregulation (10). Recent evidence also links ERCC6L to the tumor microenvironment through its association with macrophage polarization and protein lactylation signatures (22).

### Colorectal cancer

In colorectal cancer, ERCC6L knockdown leads to cell cycle arrest at the G0/G1 phase with a corresponding reduction in the S−phase population, indicating that ERCC6L promotes CRC progression primarily through cell cycle dysregulation (24).

### Renal cell carcinoma

In renal cell carcinoma, ERCC6L signals through the MAPK pathway, as ERCC6L knockdown substantially alters the phosphorylation of p38, ERK1/2, and JNK (11). Pan-cancer analyses further reveal that ERCC6L expression positively correlates with cell cycle and apoptosis pathways but negatively correlates with the TSC/mTOR pathway (7).

### Laryngeal squamous cell carcinoma

In laryngeal squamous cell carcinoma, ERCC6L promotes the nuclear translocation of FOXM1, which directly binds the KIF4A promoter to create a feed-forward loop (25). ERCC6L knockdown elevates reactive oxygen species (ROS) levels, and KIF4A knockdown attenuates the tumor−promoting effects of ERCC6L overexpression, confirming the functional significance of this circuit (25).

### Cutaneous melanoma

In cutaneous melanoma, ChIP−qPCR and dual−luciferase reporter assays demonstrate that ERCC6L directly binds the PLK1 promoter to activate its transcription. The resulting ERCC6L−PLK1 axis drives aerobic glycolysis by upregulating GLUT1, LDHA, PKM2, and HK2 (15). Critically, PLK1 inhibition or glycolysis blockade reverses ERCC6L−induced oncogenic phenotypes, experimentally validating this signaling cascade (15).

### Neuroblastoma

In neuroblastoma, the direct molecular mechanisms of ERCC6L remain to be experimentally elucidated. However, its inclusion in a stemness−related prognostic model suggests potential involvement in stemness−regulatory networks (27), warranting dedicated mechanistic investigation.

## Interaction with the tumor microenvironment

The accumulating evidence positions ERCC6L as a pivotal oncogenic factor whose functional repertoire extends beyond its canonical role in chromosome segregation to the sculpting of the tumor immune microenvironment (TIME). A consistent theme emerging from pan-cancer analyses is the robust correlation between elevated ERCC6L expression and an immunosuppressive immune landscape. Lu et al. demonstrated a positive correlation between ERCC6L levels and Th2 cell infiltration across multiple cancer types (7), while a multi-omics study in breast cancer revealed that high ERCC6L expression, as part of an HRD-associated signature, predicted reduced immune cell infiltration and a more immunosuppressive phenotype (12). In gastric cancer, ERCC6L was identified together with MYB as a key prognostic gene associated with macrophage polarization and protein lactylation, providing direct evidence linking ERCC6L to specific immune cell subsets within the TME (22).

Beyond these correlative observations, ERCC6L has emerged as a component of multi-gene signatures designed to predict immunotherapy efficacy. In breast cancer, downregulation of ERCC6L was shown to promote radiosensitivity by enhancing DNA damage, suggesting that its expression levels could guide patient selection for combined radiotherapy and immunotherapy protocols (12). A multidimensional assessment of the ERCC gene family in LUAD revealed that ERCC6L and ERCC8 exhibit the most reliable predictive performance, with ERCC6L expression showing a complex relationship with tumor-infiltrating lymphocytes (TILs) (33). Collectively, these findings underscore the translational value of ERCC6L as a biomarker for stratifying patients by likelihood of response to checkpoint blockade.

Mechanistically, ERCC6L’s well-characterized role in promoting aerobic glycolysis provides a critical link to immune evasion. ERCC6L stabilizes HIF-1α by inhibiting its hydroxylation and ubiquitin-mediated degradation, thereby enhancing the Warburg effect in LUAD cells (14). In cutaneous melanoma, ERCC6L transcriptionally activates PLK1, which drives glycolysis leading to increased lactate production (15). The resulting acidic, lactate-rich microenvironment is a well-established barrier to effective anti-tumor immunity. Furthermore, ERCC6L’s recently discovered role in activating PJA2-mediated p53 degradation promotes EMT (21), a phenotype intrinsically linked to immune evasion. The convergence of these pathways suggests that ERCC6L orchestrates a coordinated program of metabolic rewiring that simultaneously fuels proliferation and shields tumors from immune surveillance.

Although direct experimental evidence linking ERCC6L to specific immune evasion mechanisms remains limited, broader mechanistic frameworks support this connection. Chromosomal instability (CIN)—a consequence of defective mitotic processes in which ERCC6L plays a central role—has been established as a key driver of both cancer progression and immune evasion, acting through the cGAS-STING pathway to reshape the tumor immune microenvironment (34). The recent discovery that RAD52 and ERCC6L have a compensatory relationship in maintaining mitotic genome stability (35) further suggests that the level of DNA damage and chromosomal instability resulting from ERCC6L dysregulation may influence the immunogenicity of tumor cells and their susceptibility to immune surveillance.

Despite these insights, several limitations warrant acknowledgment. The majority of findings on ERCC6L’s correlation with immune infiltration derive from retrospective analyses of databases such as TCGA (7), which are inherently correlative. Functional validation has been largely restricted to *in vitro* systems or xenograft models lacking a fully functional immune system (14, 15, 21). The paradoxical finding that ERCC6L could serve both as a marker of an immune-excluded phenotype and as a target for a cancer vaccine highlights the context-dependent nature of its immunomodulatory role and demands further investigation. Future research should prioritize causal mechanistic experiments using syngeneic mouse models and single-cell sequencing to map how ERCC6L overexpression alters T cell, NK cell, and macrophage function.

## ERCC6L as a prognostic biomarker and potential therapeutic target

The consistent association between ERCC6L overexpression and aggressive tumor behavior positions it as a valuable biomarker with significant clinical potential. Its utility extends beyond simple prognostic stratification to informing therapeutic strategies and serving as a direct molecular target.

As a standalone prognostic indicator, high ERCC6L expression consistently portends worse survival outcomes across multiple cancers, including HCC (17, 18), LUAD (9, 20), GC (10), and breast cancer (6, 8). It often retains independent prognostic value in multivariate analyses that include standard clinical parameters. More sophisticated models integrating ERCC6L with other variables enhance predictive power. For instance, a nomogram combining ERCC6L expression and TNM stage showed superior prognostic ability compared to TNM stage alone in HCC (17). ERCC6L is also a key component of multi-gene prognostic signatures for various cancers. In neuroblastoma, a 4-gene signature comprising ERCC6L, AHCY, STK33, and NCAN predicts patient prognosis, with ERCC6L showing the highest predictive power (AUC = 0.799) and serving as an independent factor for both overall and event-free survival in multiple external cohorts (26). ERCC6L was also incorporated into a stemness-related prognostic model for neuroblastoma (27), while a 9-gene signature in gastric cancer (23), and an 8-hub-gene panel in breast cancer (16) further reinforce the robustness of ERCC6L as a prognostic module. In cutaneous melanoma, multi-omics analyses validated ERCC6L overexpression as an independent prognostic factor for poor survival (16).

Perhaps more impactful is the emerging role of ERCC6L in predicting treatment response, particularly to radiotherapy and chemotherapy. In breast cancer, knockdown of ERCC6L markedly elevates radiation-induced DNA damage, enhances apoptosis, and induces cell cycle arrest, thereby sensitizing cells to radiotherapy (12). This identifies ERCC6L as a novel target for radiosensitization strategies. This concept has been further corroborated in lung adenocarcinoma, where PICH deficiency was demonstrated to attenuate tumor progression and disrupt the DNA damage response, reinforcing the therapeutic potential of targeting ERCC6L to overcome radioresistance across multiple cancer types (32). Notably, a recent study by Kinsella et al. revealed that the prognostic significance of ERCC6L in radiotherapy response may be sex-dependent: low ERCC6L expression was associated with improved overall survival specifically in male patients treated with radiation (HR = 3.1, p=0.036), and radiation modified ERCC6L expression in a sex-specific manner. These findings suggest that sex-stratified analysis should be incorporated into future clinical studies evaluating ERCC6L as a predictive biomarker for radiotherapy (36). Furthermore, ERCC6L expression is implicated in chemoresistance. The protein APM2 confers cisplatin (CDDP) resistance in hepatocellular and gastric cancers by upregulating ERCC6L, which is involved in DNA excision repair. Consequently, serum APM2 levels, reflective of ERCC6L upregulation, can predict sensitivity to CDDP chemotherapy (37). This link to DNA repair pathways directly connects ERCC6L to a key mechanism of platinum-based drug resistance.

The compelling functional evidence and clinical correlations make ERCC6L a promising candidate for targeted therapy. Genetic ablation or knockdown of ERCC6L potently inhibits tumor growth in preclinical models across cancer types, with minimal adverse effects on normal tissues in experimental settings, likely due to the differential dependency of rapidly dividing cancer cells on robust mitotic machinery. The embryonic lethality observed in Pich (ERCC6L) knockout mice (2) underscores its essential role in highly proliferative contexts, a vulnerability that could be exploited therapeutically in cancers. To date, no specific ERCC6L small-molecule inhibitors have entered clinical trials; however, its partner PLK1 is a well-established druggable target. The PLK1 inhibitor volasertib has been evaluated in advanced clinical trials for acute myeloid leukemia, including Phase III investigation (2), indicating that indirectly targeting the ERCC6L-PLK1 axis could be a viable strategy. Furthermore, the synthetic lethal interaction between RAD52 and ERCC6L in mitosis (35) suggests that combining ERCC6L inhibition with RAD52 targeting could represent a novel therapeutic strategy. Targeting the ERCC6L-PLK1-glycolysis axis in melanoma (15) or the ERCC6L/HIF-1α axis in lung adenocarcinoma (14) represents specific therapeutic avenues. Natural compounds like nodosin have been shown to exert anti-tumor effects partly by downregulating ERCC6L and inhibiting its downstream PI3K/AKT signaling (31), providing a proof-of-concept for pharmacological targeting. Similarly, the anesthetic agent esketamine suppresses ESCC cell malignancy and downregulates ERCC6L expression (38), hinting at drug repurposing opportunities. While specific ERCC6L inhibitors are not yet clinically available, its interactions with druggable partners like PLK1 offer immediate indirect targeting strategies.

However, several important limitations of the current therapeutic evidence must be acknowledged. The functional evidence derives primarily from *in vitro* siRNA/shRNA knockdown experiments and xenograft models, which do not fully recapitulate human tumor biology or the pharmacokinetic challenges of targeting a nuclear helicase. Pharmacological evidence is limited to a single natural compound (nodosin) and repurposing observations with esketamine, neither of which has been validated in cancer-specific clinical trials. The association with cisplatin resistance is indirect, mediated through APM2 upregulation rather than direct ERCC6L targeting. Moreover, the embryonic lethality observed in Pich knockout mice (2) raises significant safety concerns. Therefore, while ERCC6L represents a promising research direction, its clinical translation remains distant and requires substantial further investigation.

## Conclusion and future perspectives

The accumulation of evidence over the past decade firmly establishes ERCC6L as a significant oncoprotein across a broad spectrum of human cancers. From its initial characterization as a mitotic DNA helicase, research has expanded to reveal its multifaceted roles in tumorigenesis and progression. A consistent pattern emerges: ERCC6L is frequently overexpressed in malignancies due to genetic and epigenetic alterations, its high expression robustly correlates with aggressive clinicopathological features and poor patient prognosis, and it functionally drives cancer hallmarks including sustained proliferation, evasion of cell death, and activation of invasion and metastasis.

Mechanistically, ERCC6L operates at the nexus of several core cancer pathways. It acts as a potent cell cycle accelerator by modulating G1/S and G2/M checkpoints, often through interactions with key regulators like PLK1, p53/p21, and CDK/Cyclin complexes. Its partnership with KIF4A and its ability to regulate transcription factors like FOXM1 create intricate networks that sustain mitotic fidelity and transcriptional programs favoring growth. Beyond proliferation, ERCC6L activates major oncogenic signaling cascades such as PI3K/AKT and NF-κB, and recent discoveries highlight its crucial role in reprogramming cancer metabolism toward aerobic glycolysis via the stabilization of HIF-1α or transactivation of PLK1. This metabolic reprogramming not only supplies energy but may also contribute to the stemness properties of cancer cells, further fueling tumor aggressiveness and therapy resistance.

The translational implications of these findings are substantial. ERCC6L holds great promise as a prognostic biomarker, either alone or as part of multi-gene signatures, to stratify patients for more personalized management. Importantly, its role in modulating DNA damage response and repair links it directly to treatment sensitivity, particularly to radiotherapy and DNA-damaging chemotherapies like cisplatin. This positions ERCC6L not only as a predictive marker but also as a compelling therapeutic target itself. Preclinical studies demonstrating that ERCC6L inhibition suppresses tumor growth and enhances treatment efficacy provide an initial rationale for investigation of its therapeutic potential.

Of particular emerging interest is the role of ERCC6L in the tumor microenvironment. The observed correlations with Th2 cell infiltration, macrophage polarization, and reduced immune infiltration in HRD-high tumors suggest that ERCC6L may contribute to an immunosuppressive milieu. This raises the intriguing possibility that ERCC6L expression could serve as a biomarker for immunotherapy response or resistance. Understanding the interplay between ERCC6L-mediated chromosomal instability, cGAS-STING signaling, and immune evasion will be critical for evaluating ERCC6L as a predictive biomarker for immunotherapy (34). Future studies should explore whether ERCC6L-high tumors are less responsive to immune checkpoint inhibitors and whether targeting ERCC6L could reinvigorate anti-tumor immunity.

Despite significant progress, several key questions and challenges remain for future research. First, while its interaction with partners like PLK1 is known, the detailed structural biology of ERCC6L and the precise mechanisms of its helicase/translocase activity in an oncogenic context require further elucidation. Second, the development of specific, potent, and clinically viable small-molecule inhibitors or degraders targeting ERCC6L is a critical unmet need. The clinical experience with PLK1 inhibitors and the synthetic lethal interaction between RAD52 and ERCC6L (35) provide valuable starting points for therapeutic development (39). Third, the functional consequences of ERCC6L in the tumor immune microenvironment warrant deeper investigation, especially given its correlation with specific immune cell subsets. Finally, large-scale prospective clinical studies are necessary to validate the prognostic and predictive utility of ERCC6L in diverse patient populations and to integrate it into clinical decision-making algorithms. As research continues to unravel the complexities of ERCC6L biology, this oncoprotein is poised to transition from a subject of bench-side discovery to a valuable asset in the clinical oncology toolkit, offering new avenues for improving cancer diagnosis, prognosis, and therapy.
