# Supplementary figures and images for: ERCC6L in human cancers: oncogenic functions, molecular mechanisms, and clinical implications as a prognostic biomarker and therapeutic target
Source: Front Oncol. 2026 Jul 1;16:1812829. doi: 10.3389/fonc.2026.1812829 (PMC13368489; doi:10.3389/fonc.2026.1812829)

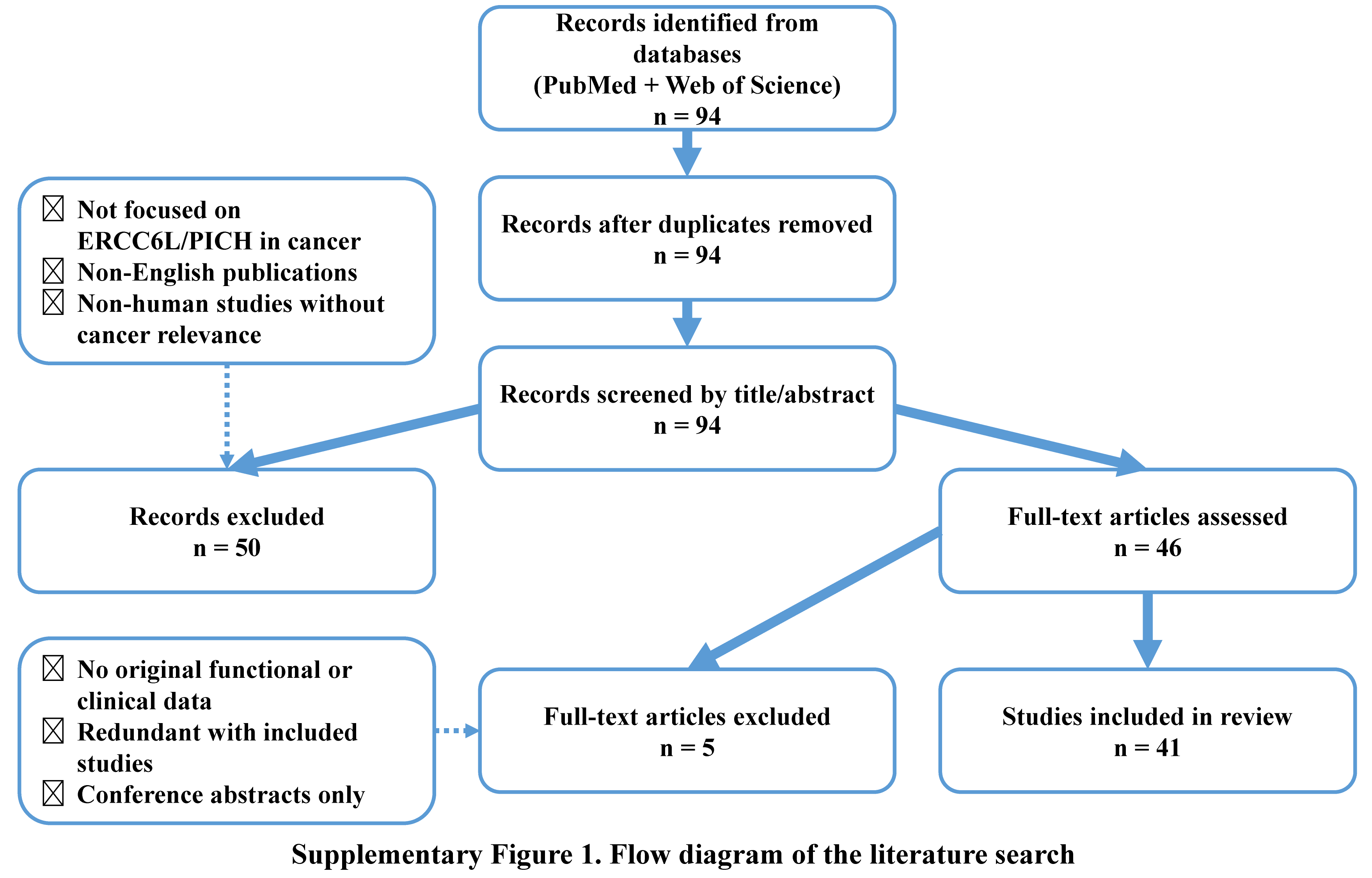

Supplement: Supplementary file 1 [file Image1.tif]
